# Comparison of silodosin to tamsulosin for medical expulsive treatment of ureteral stones: a systematic review and meta-analysis

**DOI:** 10.1007/s00240-016-0872-y

**Published:** 2016-03-28

**Authors:** Mehmet Özsoy, Evangelos Liatsikos, Nicolas Scheffbuch, Panagiotis Kallidonis

**Affiliations:** 1 Department of Urology, Comprehensive Cancer Center Vienna, General Hospital Vienna, Medical University of Vienna, Waehringer Guertel 18-20, 1090 Vienna, Austria; 2 Department of Urology, University of Patras, Rion, 26 504 Patras, Greece

**Keywords:** Medical expulsive therapy (MET), Ureteral stones, Silodosin, Tamsulosin, Meta-analysis, Systematic review

## Abstract

This study aimed at comparing the success rates of silodosin to the most commonly used for medical expulsive therapy (MET) tamsulosin for the management of ureteral stones. A systematic review using the search string: “silodosin AND (ston* OR calcu* OR expul*)” was conducted on Pubmed, SCOPUS, Web of Science, Cochrane Central Register. The Primary endpoint was the stone expulsion rate. Secondary endpoint was the time to stone expulsion. Two authors independently screened the studies depending on inclusion and exclusion criteria. Meta-analysis and forest-plot figures were calculated with the software Review Manager (RevMan 5.3.5). Variations were evaluated with the *χ* ^2^ statistical method and heterogeneity with *I* ^2^ index. After screening of 39 publications obtained by the initial search, three randomized controlled trials were eligible to be included in the meta-analysis. 407 patients were pooled. Favorable results were observed for silodosin in terms of stone expulsion rates with a risk ratio of 1.33 (95 % CI 1.17–1.50) (*I* ^2^ = 0 %). Similarly, faster stone expulsion times were observed with silodosin when compared with tamsulosin. Mean difference −2.49 (95 % CI −3.40 to 1.58) (*I* ^2^ = 89 %). This meta-analysis showed significantly higher stone expulsion rates and faster expulsion times in favor of silodosin when compared to tamsulosin.

## Introduction

Ureteral stones account for 22 % of all urinary tract stones with 68 % of them being located in the distal ureter [1]. Conservative management strategies such as observation or medical expulsive therapy (MET) using pharmacological agents to facilitate spontaneous passage of ureteral stones have gained popularity in the management of ureteral stones during the recent years [2]. Evidence on the association of stone size with spontaneous stone passage rates is scarce. 95 % of stones up to 4 mm are estimated to pass within 40 days. Moreover, stones <10 mm can be considered to pass spontaneously [3]. There is a growing body of evidence on the clinical benefit of the α-blockers in the patients with distal ureteral calculi. Specifically, the administration of α-blockers is related to a higher stone expulsion rate and shorter time periods for stone passage when compared to observation [1, 4].

The mechanism of action behind the above effects is associated with the presence of adrenergic receptors (ARs) in the ureteric smooth muscle cells with the α1-adrenergic receptors to be the most abundant [5]. α1A-, α1B- and α1D-ARs are the three types of α1-ARs that are expressed in the human ureter with the following order of abundance α1D > α1A > α1B. The blocking of these receptors results in selective relaxation of the ureteric smooth muscle and, therefore, causes ureteric lumen dilatation. The latter phenomenon results in facilitation of stone expulsion. [6–8].

The most commonly used α-blocker for MET is tamsulosin, but similar effects have been shown by other α-blockers such as terazosin and doxazosin, indicating a possible class effect [3]. Silodosin has been also proposed for MET instead of tamsulosin but studies comparing these substances for MET are scarce. In this systematic review and meta-analysis we aim to review current literature and compare the success rates of silodosin to tamsulosin for MET of ureteral stones.

## Materials and methods

### Search strategy and study selection

A systematic review using the search string; “silodosin AND (ston* OR calcu* OR expul*)” was conducted on Pubmed, SCOPUS, Web of Science, Cochrane Central Register. No restrictions were placed on language or type of publication. The search took place in February 2015. Eligibility criteria for the meta-analysis are shown in Table 1. The PRISMA statement was followed for the conduction of the study. The Primary endpoint for the meta-analysis was the stone expulsion rate. Secondary endpoint was the time to stone expulsion.

**Table 1:** Inclusion and exclusion criteria

Inclusion criteria
Ureteral stones
RCTs and MET
Comparison silodosin vs tamsulosin
Follow-up at least 14 days
Exclusion criteria
Kidney and bladder stones
Asymmetrical co-interventions (i.e. swl, JJ stent)
No comparison arm
Abstracts
Animal studies
Follow-up <14 days

### Data extraction

Two authors independently (M. Ö. and P. K.) screened the studies and extracted information on study characteristics and outcomes. Several parameters were considered for data extraction. When data were missing, the authors were contacted by email and additional data were requested.

### Validity assessment

The quality of the studies, which were included in the meta-analysis, was assessed using the Jadad score [9].

### Statistical analysis

Pooling of data for the meta-analysis took place for sildosin compared to tamsulosin. The dichotomous data for each of the eligible studies were inserted in 2 × 2 table and expressed in the form of odds ratio (OR) or mean difference with 95 % confidence intervals (CI). Data continuous in nature were pooled across studies and the weighted mean difference was calculated (WMD) with 95 % CI. The inverse variance method was used for the combination of the above result [10]. For the pooling of data characterized by a random effects model, the Der Simonian and Laird method was used [11].

Meta-analysis and forest-plot figures were calculated with the software Review Manager (RevMan 5.3.5) of the Cochrane Collaboration. Variations among the studies were evaluated using the *χ* ^2^ statistical method. *I* ^2^ index was calculated to show the proportion of inconsistency among the studies that could not be attributed to chance. Values ≥50 % were considered as significant heterogeneity [12]. A fixed effect model was used when the statistical heterogeneity did not achieve significance while a random effects model was used in the case of high heterogeneity. Statistical significance was set at *p* values of 0.05. The publication bias was assessed with the use of funnel plots and the Egger’s test [13, 14].

## Results

### Selection of studies

**Fig. 1:**
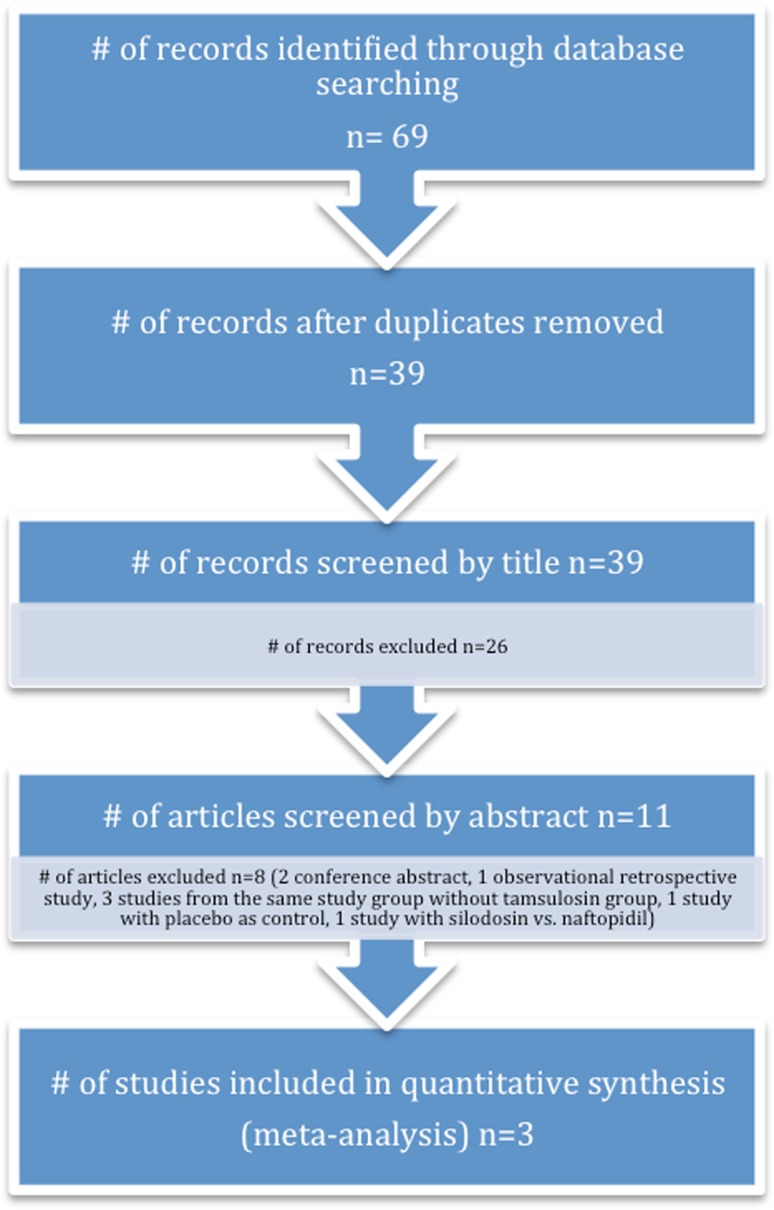
Study design

After eliminating duplicates, two aforementioned investigators screened an initial number of 39 publications by their title according to eligibility criteria. Discrepancies were resolved by consensus among the investigators. 11 publications, which passed the first elimination, were then screened by their abstracts according to the eligibility criteria. Eight studies were excluded (two conference abstract, one observational retrospective study, three studies from the same study group without tamsulosin group, one study with placebo as control group and one study with silodosin compared to naftopidil after shock wave lithotripsy therapy). Finally, only three randomized controlled trials (RCT) were eligible to be included in the meta-analysis (Fig. 1).

### Study characteristics

The three RCTs that were eligible for the meta-analysis were published between 2013 and 2014. In all of these studies a daily dosage of 8 mg of silodosin was compared with a daily dosage of 0.4 mg of tamsulosin. Two of these studies were double-blinded. Study design and treatment protocol characteristics as well as study results are described in Table 2. A detailed description of side effects was reported in two studies (Table 3).

**Table 2:** Study characteristics

Study	Inclusion criteria	Exclusion criteria	Stone location								
Study characteristics											
Dell’Atti [15]	Single lower ureteral stone, 4–10 mm, 18 years or older	Patients with severe hydronephrosis, urinary tract infection, fever, bilateral ureteral stones, solitary kidney, extra stone in the upper urinary system, previous surgical history on the ipsilateral ureter, pregnancy and/or chronic diseases	Lower ureteral								
Kumar [16]	>18 years, stone size 5–10 mm, diagnosed via NCCT or KUB, no pain after 1 day	Fever, hydronephrosis, acute or chronic renal insufficiency, multiple ureteral stones, open surgery or endoscopic interventions, diabetes, peptic ulcers, beta-blocker treatment, pregnancy, patients who demanded immediate intervention	Distal ureteral								
Gupta [17]	unilateral, non-impacted, uncomplicated middle or lower ureteral stones ≤10 mm	Not mentioned	Middle or lower ureteral								

Study	Country of origin, journal	Study period	# of patients	Randomization method	Allocation concealment	Blinding	Power analysis	Primary outcome assessed	Financial support	Authors contacted	Jadad score
Study design characteristics											
Dell’Atti [15]	Italy, Urologia	May 2012–December 2013	136	Random table envelope method	Not mentioned	Yes, blinded	No	Yes	No	Yes	3
Kumar [16]	India, Urology	January 2011–December 2012	180	Computer generated random number table	Mentioned	Yes, double blinded	Yes	Yes	No	Yes	5
Gupta [17]	India, Journal of Clinical and Diagnostic Research	February 2012–August 2012	100	Random number table	Not mentioned	Yes, double blinded	No	Yes	No	Yes	3

Study	Type of MET	Dose of MET	Duration of MET (weeks)	Type of concomitant treatment	Follow-up period (weeks)	Drop-out rate	Imaging for outcome assessment				
Treatment protocol characteristics											
Dell’Atti [15]	Silodosin/Tamsulosin	8 mg daily/0.4 mg daily	3	Diclofenac 100 mg or Paracetamol 1000 mg or Tramadol 100 mg on demand	3	3/136	X-ray, ultrasound or NCCT				
Kumar [16]	Silodosin/Tamsulosin	8 mg daily/0.4 mg daily	4	Diclofenac 50 mg on demand	4	6/174	Ultrasound and NCCT				
Gupta [17]	Silodosin/Tamsulosin	8 mg daily/0.4 mg daily	4	Diclofenac 100 mg on demand	4	0/100	X-ray, ultrasound or NCCT

**Table 3:** Side effects

Type of MET	Study			Study		
	Dell’Atti [15]	Dell’Atti [15]	*p*	Kumar [16]	Kumar [16]	*p*
	Silodosin	Tamsulosin		Silodosin	Tamsulosin	
Side effects						
Retrograde ejaculation	22.7 % (10/44)	10.2 % (4/39)	<0.00	15.6 (10/64)	11.2 (7/62)	ns
Orthostatic hypotension	3 % (2/66)	1.5 % (1/67)	ns	3.3 %	6.6 %	ns
Headache	1.5 % (1/66)	1.5 % (1/67)	ns	12.2 %	10.0 %	ns
Dizziness	6 % (4/66)	4.5 % (3/67)	ns	8.8 %	10 %	ns

*ns* not-significant

### Quality assessment of the studies

Jadad score calculations depending on randomization, blinding and definition of dropouts revealed jaded score >3 for all of the three RCTs.

### Description of the included studies results

All three authors responded to our requests, but unfortunately data especially on side effects profile and number of colic episodes were not available for all studies.

Dell’Atti et al. [15] enrolled 136 consecutive patients with solitary lower ureteral stones. Patients were randomized into two groups receiving either silodosin or tamsulosin. Four patients dropped out due to orthostatic hypotension after 1 week of medication and were excluded from the statistical analysis. After contacting the authors we have learned that ureteroscopic stone removal was performed on these dropout patients. The silodosin group showed an expulsion rate of 80.3 % whereas the tamsulosin group showed an expulsion rate of 61.2 % (*p* = 0.003). In the silodosin group, 25 patients (47.1 %) passed their stones within the first week of treatment, 21 patients (39.7 %) within 2 weeks and seven patients (13.2 %) within 3 weeks of treatment. 13 (31.7 %), 16 (39 %) and 12 (29.3 %) patients passed their stones in the tamsulosin group within 1, 2 and 3 weeks, respectively, resulting in a significant advantage in favor of silodosin (*p* = 0.002).

The second RCT, which was included in the meta-analysis consisted of three groups with 90 patients each [16]. 270 patients were randomized to receive 8 mg of silodosin, 0.4 mg of tamsulosin or 10 mg of tadalafil. Only data regarding the silodosin and tamsulosin groups were included in the analysis. The stone expulsion rate was 83.3 % in the silodosin group and 64.4 % in the tamsulosin group. Silodosin showed superior stone expulsion rates when compared with tamsulosin (83.3 vs 64.4 % *p* = 0.006) [16].

In the third RCT, 100 patients with unilateral uncomplicated middle or lower ureteral stones ≤1 cm were enrolled and randomized into silodosin and tamsulosin groups [17]. The silodosin group had significantly higher stone expulsion rates after a follow-up of 4 weeks when compared to tamsulosin group, 41/50 (82 %) patients and 29/50 (58 %) patients, respectively (*p* = 0.008). There was also a significant difference between the two groups in terms of mean stone expulsion time with 12.5 ± 3.5 vs. 19.5 ± 7.5 days in silodosin and tamsulosin groups, respectively (*p* = 0.01).

### Quantitative analysis

**Fig. 2:**
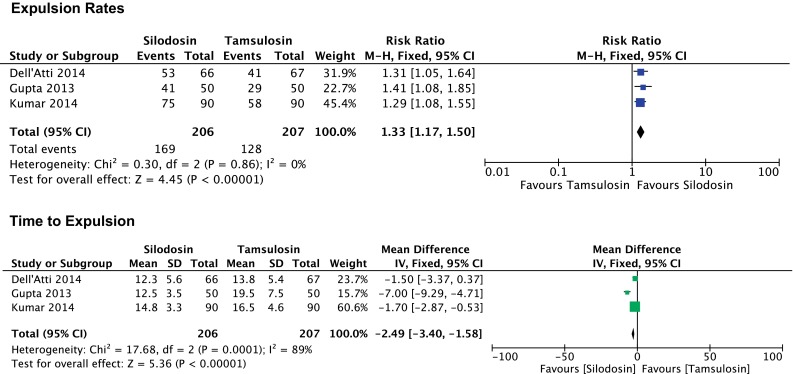
Forest plot analysis

The current meta-analysis included 407 pooled patients after the elimination of the dropouts. The pooling of the data showed favorable results for silodosin in terms of stone expulsion rates with a risk ratio of 1.33 (95 % CI 1.17, 1.50) and lack of heterogeneity (*I* ^2^ = 0 %). Patients, who were treated with silodosin, had faster stone expulsion times when compared with tamsulosin. Mean difference −2.49 (95 % CI: −3.40, −1.58) for the silodosin group. Nevertheless, the heterogeneity of the studies included in the pooled analysis was high (*I* ^2^ = 89 %) (Fig. 2).

## Discussion

Tamsulosin is a selective α1-blocker with tenfold greater affinity to α1A- and α1D- AR subtypes in comparison to α1B -AR subtype, whereas the affinity of silodosin to α1A-AR subtype is about 162-fold and 50-fold greater than its affinity to α1B- and α1D-AR subtypes, respectively [18]. Silodosin has shown superior results in the treatment of benign hyperplasia of the prostate when compared with tamsulosin in terms of efficacy and safety [19]. The above differences in the affinity of the tamsulosin and silodosin to the respective receptors may be related to the observed differences in the clinical outcome of these pharmaceutical substances for MET.

The expulsive effect for ureteral stones of silodosin has been proposed in a RCT comparing silodosin to observation treatment [20]. 112 patients were randomized into 8 mg of silodosin or observation. Both groups were directed to take 2 L of water/day. Silodosin showed superior expulsion rates especially for stones ≥5 mm. The stones of 17.9 % (*n* = 28) of the patients of the observation group and 75.9 % (*n* = 29) of the silodosin group were successfully expelled (*p* = 0.001). The expulsion time was 13.40 ± 5.90 and 9.29 ± 5.91 days, respectively (*p* = 0.012). Analgesics were required 1.5 ± 3.1 and 0.3 ± 0.9 times, respectively (*p* = 0.382). The stone size of the expulsion cases showed significant difference with 3.64 ± 1.25 and 5.23 ± 2.32 mm for the observation and silodosin groups, respectively (*p* = 0.003). The authors concluded that the administration of silodosin facilitated the expulsion of 1.5 mm or larger distal ureteral stones, as compared to the control group which did not include any medical intervention for stone expulsion [20].

The available evidence in the literature regarding the superiority of silodosin over tamsulosin for the MET of ureteral stones has been controversial. One of the studies that compare the effect of silodosin to the most commonly used substance for MET was reported by Imperatore et al. [21] The investigators retrospectively collected observational data from patients who received either silodosin or tamsulosin within a period of 1 year. The authors found no significant difference between the two groups in terms of stone expulsion rates (88 and 82 %, respectively) or mean stone expulsion times (6.7 and 6.5 days, respectively). Nevertheless, the inclusion of 50 patients in each group, despite the retrospective nature of this study, raised the question of a possible selection bias. Due to its retrospective nature, the above study was not included in the current meta-analysis.

Another study not included in the current meta-analysis was conducted by Rathi et al. [22] The study was presented as an abstract in an International Congress. The investigators randomized 87 patients with distal ureteral stones of <10 mm into three groups. Group I (*n* = 29) received 8 mg silodosin daily, group II (*n* = 30) received 0.4 mg tamsulosin daily and group III patients (*n* = 28) were not given any of the above substances. Patients in all groups received diclofenac sodium regularly for 1 week and then on demand. The follow-up period was 4 weeks. The stone expulsion rates for groups I, II and III were 86.2, 76.6 and 50 %, respectively. The difference in groups I and II with respect to group III was significant (*p* = 0.0028 and 0.035, respectively). The expulsion time was significantly shorter in groups I and II than in group III (*p* = 0.0097 and 0.026, respectively). Patients taking silodosin and tamsulosin had fewer pain attacks than group III patients. No side effects were reported in silodosin or tamsulosin groups.

The authors concluded the use of α-blockers for MET of lower ureteric stones to be safe and effective without any significant benefit of silodosin over tamsulosin.

On contrast to the above studies, the current meta-analysis showed superior stone expulsion rates and faster stone expulsion times for silodosin when compared with tamsulosin. Our pooled data showed a lack of heterogeneity for the expulsion rate calculations with an *I* ^2^ value of 0 % indicating reliable results. On the other hand, a high level of heterogeneity was calculated for stone expulsion times with an *I* ^2^ value of 89 %, but *I* ^2^ can be misleading as the magnitude and directions of the effects may influence its value and the *p* value from the *χ* ^2^ test or the CI may be related to strength of evidence of heterogeneity. When the *p* value of the *χ* ^2^ (*p* < 0.0001) and the CI [−2.49 (−3.40, −1.58)] are considered, it is clear that the expulsion time is shorter for silodosin in the current analysis. Moreover, the quality of the included studies was high according to the Jadad score (>3). When considering the low heterogeneity and the quality of the included studies, the presented results in favor of silodosin should probably be considered as reliable and accurate.

A similar meta-analysis was published recently evaluating the efficacy and safety of silodosin for MET [23]. In contrast to the current meta-anlaysis, seven studies were included. Two of the included studies were conducted by the same working group (Itoh et al.) and were published with a difference of 2 years. The investigators did not indicate in these two studies the time period for patient recruitment and it is likely that these studies may be overlapping. In addition, the aforementioned study by Imperatore et al. is not a RCT but a retrospective observational study which reduces the quality of the included studies. As a result, we could advocate that the current meta-analysis provided evidence of higher quality despite the low number of studies which were included. In fact, the latter issue is probably the major drawback of the current meta-analysis. Nevertheless, the low level of heterogeneity detected by *I* ^2^ test, especially for the primary endpoint, renders the current data to be representative. Larger scale RCTs are necessary for the confirmation of the current findings.

An important aspect of MET is the effect in the reduction of colic episodes. Kumar et al. [16] advised their patients to receive 50 mg of diclofenac on demand during MET. The mean number of pain episodes for the silodosin group was significantly lower than that for the tamsulosin group, 0.8 (SD ± 0.9) and 1.7 (SD ± 1.2), respectively (*p* < 0.001). A significantly lower requirement of analgesia was noted for silodosin (mean = 195 ± 10.2 mg) in comparison to tamsulosin (mean = 220 ± 10.8 mg) (*p* < 0.001). Similarly, Gupta et al. [17] was able to demonstrate lower analgesic use for the silodosin group. On the contrary, Imperatore et al. [21] showed no significant difference in terms of mean number of pain episodes and need for analgesics while Dell’atti et al. [15] reported infrequent and mild colic episodes in both groups that were manageable with analgesics that allowed continuation of MET. Thus, a possible but not clear reduction of colic events could be advocated for silodosin in comparison to tamsulsosin.

The evaluation of side effects is an important aspect of any medical therapy. In the case of silodosin and tamsulosin, abnormal ejaculation was the most predominant side effect observed for both silodosin and tamsulosin [15–17]. This difference was significant in the study of Dell’Atti et al. [15] which showed a significantly higher incidence of abnormal ejaculation in the silodosin group in comparison to the tamsulosin group with 22.7 and 10.2 % of the patients having experienced the side effect, respectively (*p* < 0.002). Imparatore et al. [21] also demonstrated a significantly higher incidence of abnormal ejaculation in the silodosin group when compared to tamsulosin group (*p* < 0.05). In the remaining studies, the difference among the two substances did not reach any significance. Other common side effects for both groups were orthostatic hypotension, headache, dizziness and diarrhea without any statistical difference or clinical consequence [15–17]. On the other hand, a significantly lower incidence of peripheral vasodilatation-related complications was observed for the silodosin group when compared to the patients who received tamsulosin [17, 21]. Considering the above evidence, it seems that the use of silodosin may be related to a higher incidence of ejaculation disturbances in comparison to the use of tamsulosin without any other significant difference in the side effects. The ejaculatory issues represent side effects that are reversible after the withdrawal from the MET and may not compromise the general health of the patients. Thus, the use of either substances for MET could be considered as safe.

## Conclusion

The current meta-analysis showed significantly higher stone expulsion rates and faster expulsion times in favor of silodosin when compared with tamsulosin. Both of these medications demonstrated a good safety and tolerability profile for MET in patients with uncomplicated ureteral stones. Further RCTs would strengthen the presented evidence.
